# Sensing Host Arginine Is Essential for *Leishmania* Parasites’ Intracellular Development

**DOI:** 10.1128/mBio.02023-20

**Published:** 2020-10-13

**Authors:** Adele Goldman-Pinkovich, Sriram Kannan, Roni Nitzan-Koren, Madhu Puri, Harsh Pawar, Yael Bar-Avraham, Jacquelyn McDonald, Aakash Sur, Wen-Wei Zhang, Greg Matlashewski, Rentala Madhubala, Shulamit Michaeli, Peter J. Myler, Dan Zilberstein

**Affiliations:** a Faculty of Biology, Technion-Israel Institute of Technology, Haifa, Israel; b The Mina and Everard Goodman Faculty of Life Sciences, Bar-Ilan University, Ramat-Gan, Israel; c Advanced Materials and Nanotechnology Institute, Bar-Ilan University, Ramat-Gan, Israel; d School of Life Sciences, Jawaharlal Nehru University, New Delhi, India; e Center for Global Infectious Disease Research, Seattle Children’s Research Institute, Seattle, Washington, USA; f Department of Biomedical Informatics and Medical Education, University of Washington, Seattle, Washington, USA; g Department of Microbiology and Immunology, McGill University, Montreal, Canada; h Department of Pediatrics, University of Washington, Seattle, Washington, USA; University of Geneva

**Keywords:** host-pathogen interaction, *Leishmania*, amino acid sensing, amino acid transport, intracellular parasitism

## Abstract

Arginine homeostasis in lysosomes is critical for the growth and metabolism of mammalian cells. Phagolysosomes of macrophages are the niche where the parasitic protozoan *Leishmania* resides and causes human leishmaniasis. During infection, parasites encounter arginine deprivation, which is monitored by a sensor on the parasite cell surface. The sensor promptly activates a mitogen-activated protein kinase 2 (MAPK2)-mediated arginine deprivation response (ADR) pathway, resulting in upregulating the abundance and activity of the *Leishmania* arginine transporter (AAP3). Significantly, the ADR is also activated during macrophage infection, implying that arginine levels within the host phagolysosome are limiting for growth. We hypothesize that ADR-mediated upregulation of AAP3 activity is necessary to withstand arginine starvation, suggesting that the ADR is essential for parasite intracellular development. CRISPR/Cas9-mediated disruption of the *AAP3* locus yielded mutants that retain a basal level of arginine transport but lack the ability to respond to arginine starvation. While these mutants grow normally in culture, they were impaired in their ability to develop inside THP-1 macrophages and were ∼70 to 80% less infective in BALB/c mice. Hence, inside the host macrophage, *Leishmania* must overcome the arginine “hunger games” by upregulating the transport of arginine via the ADR. We show that the ability to monitor and respond to changes in host metabolite levels is essential for pathogenesis.

## Abstract (precis)

In this study, we report that the ability of the human pathogen *Leishmania* to sense and monitor the lack of arginine in the phagolysosome of the host macrophage is essential for disease development. Phagolysosomes of macrophages are the niche where *Leishmania* resides and causes human leishmaniasis. During infection, the arginine concentration in the phagolysosome decreases as part of the host innate immune response. An arginine sensor on the *Leishmania* cell surface activates an arginine deprivation response pathway that upregulates the expression of a parasite arginine transporter (AAP3). Here, we use CRISPR/Cas9-mediated disruption of the *AAP3* locus to show that this response enables *Leishmania* parasites to successfully compete with the host macrophage in the “hunger games” for arginine.

## INTRODUCTION

Protozoan parasites of the genus *Leishmania* are the causative agents of a wide spectrum of human and veterinary diseases. *Leishmania* species cause morbidity and mortality throughout the Old and New Worlds, with clinical manifestations ranging from lesions of the skin (cutaneous leishmaniasis [CL]) and mucous membranes (mucocutaneous leishmaniasis [MCL]) to lethal infection of the spleen and liver (visceral leishmaniasis [VL]) (1). Approximately 350 million people in 88 countries are at risk of VL, with as many as 500,000 new cases diagnosed every year, 10% of which are fatal (2).

Leishmania donovani, the causative agent of kala-azar (VL), exhibits a digenetic life cycle that includes both insect and mammalian forms. Extracellular promastigotes develop in the alimentary tract of sand flies. Following infection of the mammalian host, promastigotes differentiate into intracellular amastigotes within the phagolysosome of macrophages (3, 4). This differentiation process in the host can be mimicked in axenic cultures by shifting promastigotes from an insect-like (26°C and pH 7) to an intralysosomal (37°C, pH 5.5, and 5% CO_2_) environment (5–7).

During infection, *Leishmania* parasites encounter macrophage defense mechanisms designed to interdict parasite invasion and block their intracellular survival, including the release of reactive oxygen species (ROS) and the synthesis of cytotoxic nitric oxide (NO) by inducible nitric oxide synthase (iNOS). Yet for macrophages to produce effective amounts of NO, they must import arginine from the extracellular environment. Countering the host NO attack, parasitic invasion activates macrophage arginase 1, which converts arginine to ornithine, the first substrate of the polyamine pathway, thereby suppressing NO synthesis and promoting parasite survival (8). However, the reduction of the host arginine pool becomes a double-edged sword for *Leishmania* parasites in infected macrophages since they cannot synthesize arginine *de novo*. Instead, they must import exogenous arginine via a monospecific amino acid transporter (AAP3) (9), using it primarily in the polyamine pathway to provide precursors for trypanothione biosynthesis (10). Thus, they encounter an intriguing metabolic dilemma: on the one hand, emptying the host arginine pool provides an advantage (reduction of NO), but at the same time, it causes a potentially deadly disadvantage by blocking the supply of an essential amino acid (8). Therefore, to survive, *Leishmania* parasites need to sense and respond to changes in arginine availability.

A few years ago, we discovered that upon arginine starvation, *Leishmania* parasites promptly activate a mitogen-activated protein kinase 2 (MAPK2)-mediated arginine deprivation response (ADR) pathway, resulting in the upregulation of the expression and activity of the *Leishmania* arginine transporter (AAP3) (11). Significantly, the ADR is also activated during macrophage infection due to intracellular parasites actively depleting arginine within the host phagolysosome (12).

The L. donovani genome contains two (haploid) *AAP3* gene copies that are tandemly arrayed on chromosome 31 (chr31), which is tetrasomic in L. donovani (and most other *Leishmania* species). While the coding sequences (CDSs) of the *AAP3.1* and *AAP3.2* genes are (almost) identical, their 3′ untranslated regions (UTRs) are quite different, and only (4.5-kb and 3.5-kb) mRNAs from the latter (*AAP3.2*) are upregulated under conditions of arginine deprivation (10). Genome-scale transcriptomic analysis (RNAseq) revealed only five other changes in gene expression (mostly other transporters) associated with arginine deprivation, indicating the presence of a coordinated ADR (11).

Nutrient sensing is an essential means for parasites to adapt to and successfully settle inside the vector and host (13). Studies by several laboratories have recently identified intriguing nutrient sense response mechanisms in *Leishmania*. Martin et al. (14) reported that intracellular sensing of purine starvation directs promastigotes into long-term cell cycle arrest at G_1_-G_0_ until a new supply of purine is provided. Starvation of Leishmania mexicana promastigotes for glucose induced a 50-fold increase in the abundance of LmGT1, a glucose transporter that localizes to the flagellum (15). In the case of the facultative intracellular parasite *Leishmania*, arginine availability is of particular interest since it is important for both host defense and parasite proliferation. Sensing of arginine levels in the lysosome lumen is a key mechanism that regulates mTORC1 activity in mammalian cells (16). In macrophages, mTORC1 activation induces a Th1 response (17). This pathway is the major means by which macrophages kill invading pathogenic microorganisms. Interestingly, *Leishmania* parasites are able to counteract this outcome by activating a Th2 response, directing arginine toward polyamine biosynthesis instead of NO production, thereby enabling the parasites to persist and cause long-term nonhealing infections (18, 19). However, both Th1 and Th2 responses result in arginine depletion in the macrophage phagolysosome, presenting an existential threat to parasite survival since they are unable to synthesize this essential amino acid.

In this study, we explore the mechanism that enables intracellular *Leishmania* to win this “hunger game.” Using CRISPR/Cas9, we created mutants that lack AAP3.2 and thereby are unable to upregulate *AAP3* expression after arginine starvation. These mutants were unable to grow in either THP-1 macrophages or BALB/c mouse livers. This study shows that sensing host nutrients is essential for intracellular parasite development.

## RESULTS AND DISCUSSION

The *Leishmania* genome contains two genes (*AAP3.1* and *AAP3.2*) in a tandem array on chromosome 31 that encode arginine transporters. They are highly conserved (only 3 amino acid differences) within their CDSs and 5′ UTRs but have very different 3′ UTRs. However, only *AAP3.2* is responsive to arginine deprivation (10). To assess whether the *AAP3.2* response to arginine deprivation is necessary for parasite intracellular development, we aimed to delete it from the L. donovani genome. Unfortunately, chromosome 31 is tetraploid in L. donovani (20), making classical homologous gene replacement approaches cumbersome and tedious, especially since the plasticity of the *Leishmania* genome enables parasites to retain an additional wild-type (WT) chromosome as well as those containing the selectable marker(s) (21). Therefore, we used the *Leishmania*-adapted CRISPR/Cas9 system (22) to expedite the disruption of the AAP3 genes.

**FIG 1:**
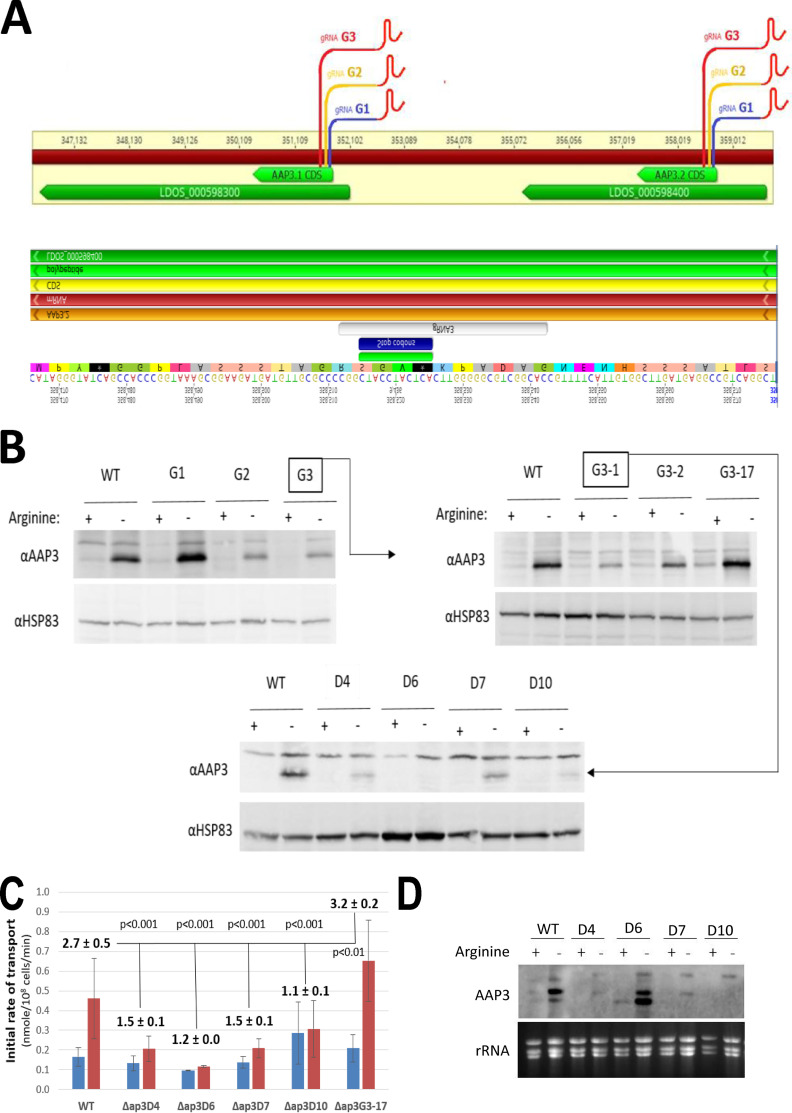
Targeting CRISPR guides to the 5′ end of the *AAP3* open reading frame (ORF) impairs their response to arginine deprivation. (A, top) *Leishmania*-adapted CRISPR/Cas9 targeted to the 5′ end of the *AAP3* ORF at bp 19, 85, and 173 in the CDS with gRNAs designated G1, G2, and G3, respectively. gRNA-transfected promastigotes were grown in cultures supplemented with neomycin (50 μg/ml) for 6 weeks. (B, top left) Subsequently, proteins extracted from these cells were subjected to Western analysis using anti-LdAAP3 antiserum (and HSP83 antiserum for a loading control). (B, top right) G3 cells (highlighted in squares) were then seeded on agar plates, and three types of colonies were raised, Δ*ap3G3-1*, Δ*ap3G3-2*, and Δ*ap3G3-17*. (A, bottom) The Δ*ap3G3-1* mutant (highlighted in a square) was then further transfected with a donor, i.e., the gRNA G3-based sequence containing an 11-bp insertion with stop codons. (B, bottom) These transfectants gave rise to several colonies of Δ(*aa3-adr*) mutants that we named Δ*ap3D4*, Δ*ap3D6*, Δ*ap3D7*, and Δ*ap3D10*. (C) Fold change of the initial rate of arginine transport (2 min) (see Fig. S1 in the supplemental material) before and after 2 h of arginine starvation (blue and red, respectively). (D) Northern analysis of AAP3 mRNA expression before and after 2 h of arginine starvation.

WT L. donovani was transfected (separately) with CRISPR plasmids containing 21-nucleotide (nt) guide RNAs (gRNAs) (G1, G2, and G3) targeting the 5′ ends (positions +19, 85, and 173) of both the *AAP3.1* and *AAP3.2* CDSs (Fig. 1A, top), and the cultures were examined 6 weeks later for AAP3 protein levels in the presence or absence of arginine. While the G1-transfected cultures showed enhanced ADR-mediated increases in AAP3 levels compared to the WT, the G2 and G3 cultures showed a diminished capacity to upregulate AAP3 levels after arginine starvation (Fig. 1B, top left). The G3 culture was seeded onto agar plates, and individual colonies were examined for an ADR, with different clones showing normal (Δ*ap3G3-2*), enhanced (Δ*ap3G3-17*), or reduced (Δ*ap3G3-1*) increases in AAP3 protein abundance compared to the WT (Fig. 1B, top right). In order to increase the efficiency of CRISPR/Cas9-mediated gene disruption, the Δ*ap3G3-1* mutant was transfected (four times at 3-day intervals) with a 61-nt “donor” oligonucleotide consisting of an 11-nt insertion with a stop codon in all frames and 25-nt sequences flanking the Cas9 cleavage site (Fig. 1A, bottom). Single colonies were isolated 3 weeks after transfection and examined for AAP3 expression levels after arginine deprivation. Two clones (Δ*ap3D6* and Δ*ap3D10*, D6 and D10, respectively) showed no increase in AAP3 protein abundance after arginine deprivation, while two others (Δ*ap3D4* and -*D7*, D4 and D7, respectively) showed a small (less than WT) increase in AAP3 protein levels (Fig. 1B, bottom). Analyses of the initial rates of arginine transport confirmed that these mutants had lost all or some of their response to arginine deprivation (Fig. 1C; see also Fig. S1 in the supplemental material). While WT cells showed a 2.7-fold increase in the initial rate of arginine transport 2 h after arginine starvation, most of the mutants showed little or no increase in the transport rate after arginine starvation (1.5-, 1.2-, 1.5-, and 1.1-fold for Δ*ap3D4*, Δ*ap3D6*, Δ*ap3D7*, and Δ*ap3D10*, respectively). Interestingly, the Δ*ap3G3-17* mutant showed a 3.2-fold (larger than WT) increase in transport after arginine deprivation, consistent with additional copies of the *AAP3.2* gene (see below).

10.1128/mBio.02023-20.1 FIG S1 Initial rates of arginine transport in wild-type and mutant L. donovani promastigotes. Two-minute arginine transport was determined using the rapid filtration technique (see Materials and Methods). Transport was assayed in the wild type before (diamond) and after (square) 2 h of arginine starvation and in the Δ*ap3D6* mutant before (diamond) and 2 h after (square) arginine starvation. Download FIG S1, EPS file, 1.0 MB. Copyright © 2020 Goldman-Pinkovich et al. 2020 Goldman-Pinkovich et al. This content is distributed under the terms of the Creative Commons Attribution 4.0 International license.

**FIG 2:**
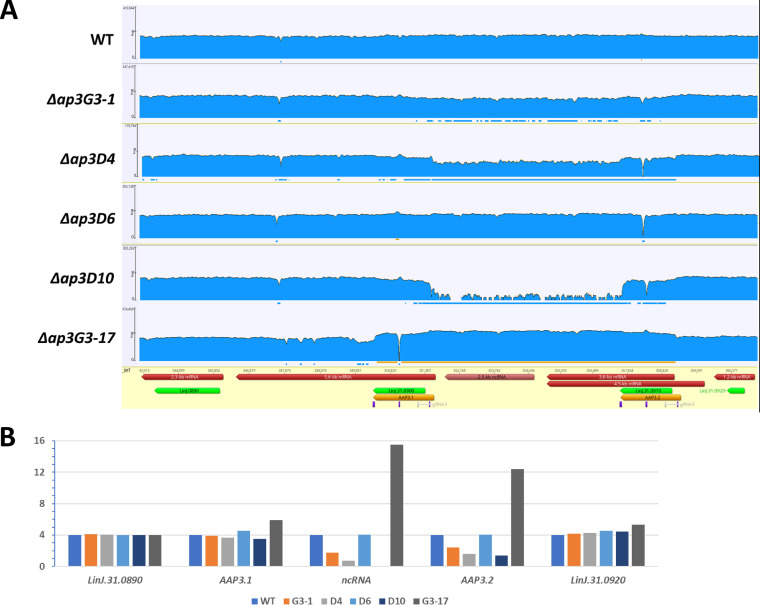
Whole-genome sequencing of the AAP3 region in CRISPR/Cas9-treated L. donovani promastigotes. Whole-genome sequencing was carried out for the WT and each of the Δ(*aap3-adr*) mutants. Illumina libraries were constructed and sequenced on a HiSeq 4000 instrument using paired-end 75-bp reads with TruSeq standard primers. (A) Reads were aligned against the L. donovani 1S reference genome and revealed that mutants Δ*ap3G3-1*, Δ*ap3D4*, and Δ*ap3D10* harbor a large deletion essentially fusing the AAP3.1 CDS with that of AAP3.2 and eliminating the AAP3.2 3′ UTR and all intergenic ncRNA sequences. Quantification of reads indicates that a gene fusion event has occurred on two copies of chromosome 31 in the Δ*ap3G3-1* mutant, on three copies in Δ*ap3D4*, and on four copies in Δ*ap3D10*. The Δ*ap3G3-17* mutant harbors 16 copies of the region. Δ*ap3D6* alignment to a version of LdoS_31 revealed an 11-bp insertion (with a stop codon in each frame) in the AAP3.2 gene. The read counts for each gene represent only those that can be unambiguously assigned to the WT or mutant version of the gene. These results are consistent with the hypothesis that 3 copies of AAP3.2 and 3 copies of the 4 AAP3.1 genes contain stop codons in the Δ*ap3D6* mutant. (B) Read analysis indicates that neighboring genes such as LinJ.31.0890 and LinJ.31.0920 were not affected by the AAP3 mutation.

Whole-genome sequencing was carried out to map the precise location of the CRISPR/Cas9-induced mutations in each cell line. Illumina libraries were constructed and sequenced on a HiSeq 4000 instrument using paired-end 75-bp reads with TruSeq standard primers. Between 45.9 million (Δ*ap3D4*) and 96.3 million (Δ*ap3G3-17*) reads were obtained per library, and they were aligned against the L. donovani 1S genome sequence assembled from a combination of PacBio and Illumina reads (A. Sur, J. R. McDonald, G. Ramasamy, and P. J. Myler, unpublished data). Changes in coverage (read counts) were seen (Fig. 2A) in the *AAP3* locus of all mutants (Δ*ap3D7* is not shown since it was essentially identical to Δ*ap3D4*). As expected, the WT parent had four copies of both *AAP3.1* and *AAP3.2* as well as the genes flanking this locus (Fig. 2B). However, the Δ*ap3G3-1* mutant and most of its progeny (*D4* and *D10*) had substantially lower coverage in the sequence between the 5′ UTR of *AAP3.1* and the 3′ end of *AAP3.2*, consistent with recombination near the double-strand break(s) introduced by Cas9 at the site of the gRNA G3 sequence to create an *AAP3.1*/*AAP3.2* fusion that contains the 5′ UTR of *AAP3.*2 and the 3′ UTR of *AAP3.1* along with a deletion of the 3′ UTR of *AAP3.*2 (and the non-coding RNA [ncRNA] located in the intergenic region between the two *AAP3* genes). Quantitation of the read counts (Fig. 2B) indicated that the Δ*ap3G3-1* mutant retained two intact copies of *AAP3.1* and *AAP3.2*, along with two copies of the *AAP3.1*/*AAP3.2* fusion, and Δ*ap3G3-1/D4* (and Δ*ap3G3-1/D7*) retained one copy of the intact *AAP3* locus (containing both *AAP3.1* and *AAP3.2*) and three copies of the *AAP3.1*/*AAP3.2* fusion, while in Δ*ap3D10*, all four copies of the *AAP3* locus contained the *AAP3.1*/*AAP3.2* fusion. A small number of reads, 102, from this region are present in the Δ*ap3D10* mutant, suggesting that some cells in this population may retain an intact copy of chr31 (or the sample was contaminated with another cell line). In contrast, the Δ*ap3G3-17* mutant showed higher coverage in this region, consistent with recombination between the two copies of the *AAP3* gene resulting in additional (2 to 3 for each copy of chr31) copies of *AAP3.2* and the intergenic sequence (including the ncRNA). Interestingly, the Δ*ap3D6* mutant shows slightly higher than WT levels of read coverage for the *AAP3.1* gene, suggesting that it retained one copy of chr31 with the *AAP3.1*/*AAP3.2* fusion along with three copies containing both the *AAP3.1* and *AAP3.2* genes (one of which may contain an additional *AAP3.2* gene). Examination of the read coverage in the gRNA G3 region indicated that 78% (418/536) contain the 11-bp insertion with a stop codon in all frames, consistent with the hypothesis that the Δ*ap3D6* mutant contained seven copies of the *AAP3.1* and *AAP3.2* genes with stop codons that render them nonfunctional at the protein level and only one (or two) functional version(s). However, the inability of this mutant to present with even a partial ADR on the protein level (such as that of Δ*ap3D4* or Δ*ap3D7*) suggests that the only functional copies without stop codons are *AAP3.1*/*AAP3.2* fusions that lack ADR capacity.

Northern analyses (Fig. 1D) confirmed the reduction in the *AAP3.2* gene copy number in the *D4*, *D7*, and *D10* cell lines by showing that its mRNA levels after arginine deprivation were significantly lower than those of the WT. These changes paralleled the changes in protein abundance observed in Fig. 1B, confirming that AAP3.2 accounts for most (if not all) of the increase in arginine transport after arginine starvation. Importantly, even though the *AAP3.2* mRNA levels in the Δ*ap3D6* mutant were similar to WT levels in both the presence and absence of arginine, AAP3 protein levels were not upregulated in response to arginine deprivation because of the stop codons in the *AAP3.2* gene. Two other ADR-responsive genes (*LinJ.10.1450* and *LinJ.36.2900*, which encode pteridine and MFS family transporters, respectively) (11) responded normally to arginine deprivation in all mutants (Fig. S2), indicating that the CRISPR/Cas9 mutations affected only the response of *AAP3.2* to arginine deprivation, not the entire ADR pathway.

10.1128/mBio.02023-20.2 FIG S2 Other members of the ADR retained sensitivity to arginine deprivation. Real-time PCR analysis was carried out to determine mRNA levels of pteridine (top) and MFS (bottom) transporters before and 2 h after arginine starvation. The analyses were carried out in Δ*ap3D4* (D4), Δ*ap3D6* (D6), Δ*ap3D7* (D7), and Δ*ap3D10* (D10) mutants. Results are relative to 2-h arginine-deprived WT cells. Download FIG S2, EPS file, 1.5 MB. Copyright © 2020 Goldman-Pinkovich et al. 2020 Goldman-Pinkovich et al. This content is distributed under the terms of the Creative Commons Attribution 4.0 International license.

**FIG 3:**
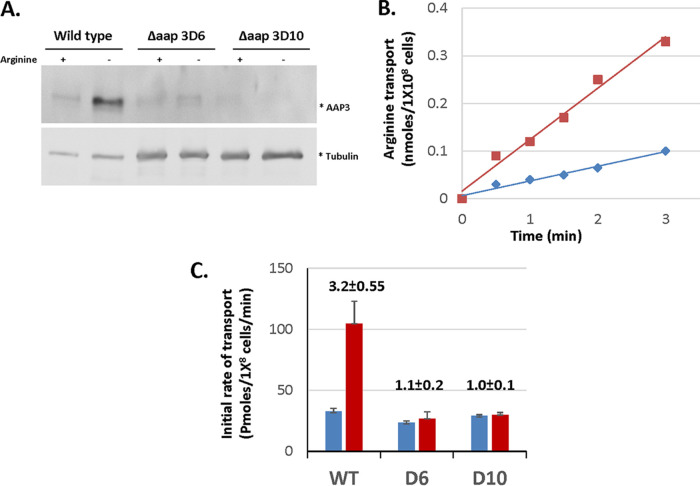
Wild-type axenic amastigotes, but not AAP3.2-null mutants, respond to arginine deprivation. Wild-type, Δ*ap3D6* (D6), and Δ*ap3D10* (D10) L. donovani amastigotes were subjected to 2 h of arginine deprivation as described in Materials and Methods. (A) A portion of each culture was subjected to SDS-PAGE, transferred to nitrocellulose, and then subjected to anti-AAP3 antibodies. (B) The other portion was subjected to analysis of the initial rate of arginine transport. (C) The slopes of each transport assay were used to calculate the rate of transport.

The analyses described above indicated that the Δ*ap3D6* and Δ*ap3D10* mutants are the most informative *AAP3* mutants, retaining near-WT basal levels of arginine transport (when grown in normal medium), but are unable to upregulate transporter activity after arginine deprivation. Therefore, we decided to conduct further studies with these two mutants to determine whether the upregulation of AAP3 expression is necessary for intracellular growth. First, we assessed whether amastigotes retain the phenotypes observed for promastigotes. Axenic promastigotes, WT and mutants, differentiated into amastigotes in culture (7). The rates of differentiation were identical in both the WT and the Δ*ap3D6* and Δ*ap3D10* mutants; i.e., they reached maturation within 5 days (6). Mature amastigotes were then subjected to 2 h of arginine starvation. As shown in Fig. 3A, WT axenic amastigotes responded to arginine deprivation by increasing the AAP3 protein abundance 2.3-fold. In contrast, Δ*ap3D6* and Δ*ap3D10* amastigotes were insensitive to arginine deprivation, as were mutant promastigotes.

In correlation with ADR-driven AAP3 expression in amastigotes, the initial rate of arginine transport in the WT increased by 3-fold following 2 h of arginine deprivation (Fig. 1B and C). Both Δ*ap3D6* and Δ*ap3D10* amastigotes transported arginine at a rate similar to that of unstarved WT amastigotes but remained unchanged after arginine deprivation. Hence, the results indicate that wild-type and mutant amastigote responses to arginine deprivation were identical to those of promastigotes. This further indicates that the rate of arginine transport in L. donovani is regulated by the abundance of the AAP3 protein.

**FIG 4:**
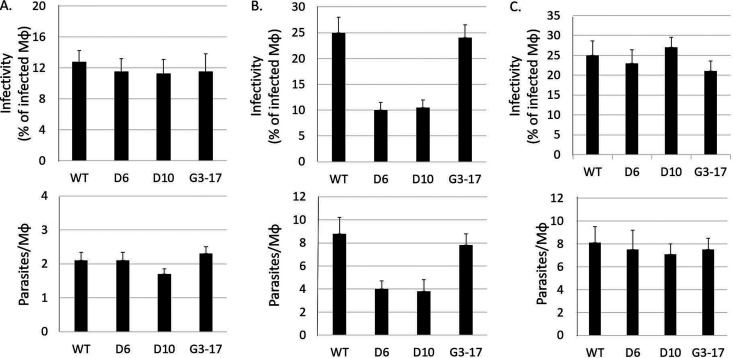
Mutants lacking the AAP3 ADR lost infectivity in THP-1 macrophages. Human THP-1 differentiated macrophages grown in RPMI 1640 medium containing 0.1 mM arginine were subjected to infection by mid-log-phase L. donovani promastigotes of the WT and Δ*ap3D6* (D6), Δ*ap3D10* (D10), and Δ*ap3G3-17* (G3-17) mutants at an MOI of 10 for 4 h, as described in Materials and Methods. Following 4 h of coincubation, extracellular parasites were washed away, and this point is referred to as time zero for the purpose of the infection start point. At time zero and 48 h after infection, macrophages were fixed, Giemsa stained, and subsequently subjected to counting. (A and B) The intracellular growth capacity was determined by calculating infectivity (top) and parasitemia (bottom). Infectivity was calculated as the percentage of infected macrophages (Mϕ) of the total counted macrophages (*n* = ≥100 cells). Parasitemia is calculated as the average number of parasites per infected macrophage. Macrophage infections were carried out in the presence of 0.1 mM (A and B) or 1.5 mM (C) arginine. For panel B, one-way ANOVA indicated that the Δ*ap3D10* and Δ*ap3D6* mutants were 57% less infective than the WT (*P* < 0.05; *n* = 16), and parasitemia was 50% lower than for the WT (*P* < 0.05; *n* = 4). (C) When infection was performed at a 1.5 mM external arginine concentration, one-way ANOVA was insignificant for mutant impairment in either infectivity (top) or parasitic burden (bottom) parameters (*P* < 0.1).

To determine whether the ADR is necessary for intracellular growth, we infected THP-1 macrophages with late-log-phase L. donovani promastigotes of the WT and Δ*ap3D6*, Δ*ap3D10*, and Δ*ap3G3-17* mutants in medium containing a physiologically relevant (0.1 mM) concentration of arginine (12, 23). As shown in Fig. 4A, the initial level of infection (i.e., the percentage of macrophages infected after 4 h of coincubation) (Fig. 4A, top) and parasite burden (i.e., the average number of parasites per infected macrophage) (bottom) with all three mutants were similar to those of the WT. However, by 48 h postinfection (Fig. 4B), the infectivity and parasite burden for both the Δ*ap3D6* and Δ*ap3D10* mutants were reduced by 2-fold or more compared to the WT, while the levels for Δ*ap3G3-17* were comparable to those of the WT. Hence, our results indicate that while the mutants can infect macrophages as well as the WT, they fail to proliferate normally thereafter. Indeed, it appears that a significant number of macrophages completely cleared their internalized parasites (since the percentage of infected macrophages was lower after 48 h than that after 4 h), while the parasites in the remaining infected macrophages underwent only 1 or 2 rounds of replication (since the parasites per macrophage increased only ∼2-fold), compared to the normal 3 to 4 rounds of replication and reinfection of new macrophages seen with WT parasites.

To assess whether the lower AAP3 expression levels (and consequently less arginine import) were responsible for the reduction in the infectivity and parasite burden of the Δ*ap3D6* and Δ*ap3D10* mutants, we infected THP-1 macrophages grown in medium containing 1.5 mM arginine, a concentration that prevented the ADR in intracellular WT amastigotes (12). Under these conditions, both the Δ*ap3D6* and Δ*ap3D10* mutants developed normally into amastigotes, with infectivity and parasitemia at 48 h similar to those of the WT (Fig. 4C). The results indicate that the inability to develop inside macrophages is due to arginine deprivation in macrophage phagolysosomes during infection and that the ability to respond to this deprivation by upregulating *AAP3.2* protein levels is essential for successful intracellular *Leishmania* development.

**FIG 5:**
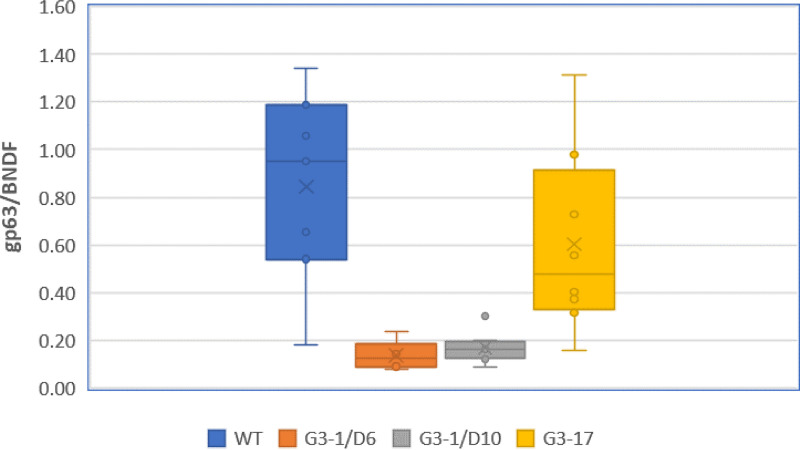
Parasites that lack *AAP3.2* are unable to develop in mice. Late-stationary-phase L. donovani promastigotes of the WT and Δ*ap3D6*, Δ*ap3D10*, and Δ*ap3G3-17* mutants were injected intravenously (i.v.) into 8-week-old female BALB/c mice (1 × 10^8^ cells per injection; eight mice per mutant). On day 21 postinfection, mice were sacrificed, and liver DNA was extracted using the proteinase K method (26). Analyses were carried out as described in Materials and Methods.

**FIG 6:**
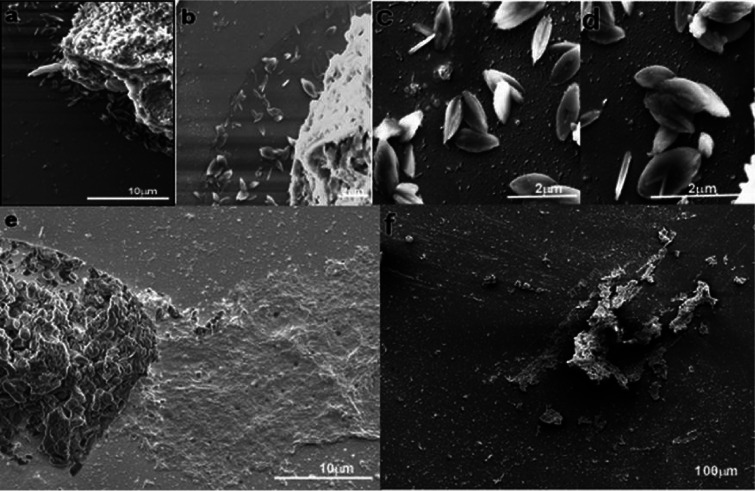
Scanning electron microscopy of crude WT and mutant L. donovani-infected liver homogenates. Dissected mouse liver samples were crushed in the presence of Karnovsky fixative to prepare a crude liver homogenate, which was processed further with tannic acid fixation and 4% osmium tetroxide. The samples were serially dehydrated in ethanol, coated with gold, and visualized using an FEI Quanta 200 FEG electron microscope at 20 kV. (a) WT-infected liver tissue piece with amastigotes seen underneath the tissue piece. (b to d) Enlarged images from the wild-type-infected liver tissue piece. (e and f) D6-infected liver tissue piece with amastigotes found negative.

To further assess the role of arginine transport *in vivo*, we infected BALB/c mice (8 per group) with Δ*ap3D6*, Δ*ap3D10*, Δ*ap3G3-17*, and WT parasites. On day 21 postinfection, the mice were sacrificed, and tissue parasite burdens were determined by quantitative PCR (qPCR) on the DNA extracted from the liver of each mouse. As shown in Fig. 5, the parasite burdens of the Δ*ap3D6* and Δ*ap3D10* mutants averaged only 20 and 24%, respectively, of that of the WT. One-way analysis of variance (ANOVA) and Tukey *post hoc* honestly significant difference (HSD) testing showed a significant difference (*P* < 0.001) from the WT for both the Δ*ap3D6* and Δ*ap3D10* mutants, while the Δ*ap3G3-17* mutant was not significantly different from the WT (*P* = 0.387). To further evaluate the level of infection, dissected mouse liver samples (*n* = 6 for each group) were crushed in the presence of Karnovsky fixative to prepare crude liver homogenates. These were fixed and subsequently subjected to scanning electron microscopy (Fig. 6). As shown, livers from mice infected with WT parasites were highly infected with amastigotes (Fig. 6a to d), while livers infected with the Δ*ap3D6* mutant were almost clear of parasites (Fig. 6e and f), supporting the qPCR data that mutant parasites that lack AAP3.2 are unable to develop colonies in their host.

These results show that the inability of the mutants to express higher levels of AAP3 in order to compensate for the reduced level of arginine in the phagolysosomes of infected macrophages severely compromised their ability to develop in the liver. Since spleens were not included in this analysis, it is possible that differential expression of arginine metabolism-related enzymes in the different organs may influence the role and significance of the AAP3-mediated ADR in parasite survival. Nevertheless, we show here that the liver as a major target of this pathogen presents with significantly lower parasite burdens for mutant parasites than for both WT and AAP3.2-overexpressing parasites (G3-17), which serve as an alternative to an add-back control. Therefore, it appears that the ADR is a crucial mechanism for enabling intracellular *Leishmania* parasites to overcome the arginine bottleneck.

In summary, this study demonstrates that the ability to monitor metabolic deprivation and subsequently induce a specific response at the level of gene expression is essential for the pathogenesis of a protozoan parasite. Furthermore, we have shown that for pathogenic microorganisms to respond to host-inflicted environmental changes and survive, they must employ external sensing and response mechanisms to serve as their monitoring device.

## MATERIALS AND METHODS

### *Leishmania* strains and culture.

L. donovani MHOM/SD/00/1S (24) promastigotes were grown in medium M-199, Earle’s salts (Biological Industries Ltd.) supplemented with 10% heat-inactivated fetal bovine serum (FBS) (Gibco Ltd.) and a 1% penicillin-streptomycin solution (Biological Industries Ltd.). Axenic differentiation of L. donovani promastigotes to amastigotes was carried out as described previously (7). Briefly, mid-log-phase promastigotes were washed twice in Earle’s salt solution and finally suspended in amastigote medium containing M-199 at pH 5.5 supplemented with 0.5 mM sodium succinate, 25% bovine serum, and 1% penicillin-streptomycin solution. Mature amastigotes developed at 5 days after exposure of promastigotes to the differentiation medium.

Arginine deprivation was carried out as described previously by Pawar et al. (12). Briefly, mid-log-phase promastigotes (1 × 10^7^ cells/ml) were washed with Earle’s balanced salt solution twice and resuspended in arginine-deficient medium M-199 (Biological Industries Ltd.) at 26°C for the specified period before being transferred to ice. Arginine-deprived cells were washed twice with ice-cold Earle’s balanced salt solution before being used for transport assays and Northern and Western blot analyses.

### CRISPR/Cas9 guide and donor transfections.

gRNA sequences were designed using the Eukaryotic Pathogen CRISPR Guide RNA/DNA Design Tool (EuPaGDT) (http://grna.ctegd.uga.edu/) and cloned into *Leishmania*-adapted vector pLdCN using the single-step digestion-ligation cloning protocol previously described (22), and the constructs were transfected into mid-log-phase promastigotes. Following gRNA-pLdCN transfections, cells were grown for 4 weeks with G418 at 50 μg/ml and subsequently screened for their ability to increase LdAAP3 protein abundance after arginine deprivation. Next, the G3 donor sequence was introduced into a gRNA G3-originated clone exhibiting the desired phenotype. The G3 donor is a single-strand oligonucleotide donor (sense) containing 25-nt sequences flanking the Cas9 cleavage site (shown in boldface type below) and an 11-nt sequence with a stop codon in all three frames (underlined and italicized below). Three transfections with 10 μl (100 μm) of this oligonucleotide were performed on promastigotes at 3-day intervals as previously described (22). gRNA sequences are as follows: GTCTATTCCAGCACAGGCGG for gRNA G1, GCCGTCGATAAACACCCGAG for gRNA G2, GTGCCGACGCCGCCAAGCCG for gRNA G3, and ATGAAAACG**GTGCCGACGCCGCCAAG***TGAGTAGGTAG***CCG**GGGCGCAACATCATCTTCCG for the G3-based donor.

### Arginine deprivation.

Arginine deprivation of axenic promastigotes and amastigotes was carried out as described previously by Goldman-Pinkovich et al. (11). Briefly, logarithmic-phase promastigotes or amastigotes (1 × 10^7^ cells/ml) were washed twice in ice-cold Earle’s salt solution at pH 7 (promastigotes) or pH 5.5 (amastigotes) and subsequently suspended to a final density of 1 × 10^7^ cells/ml in arginine-free Earle’s salt-based medium 199 (Biological Industries Ltd., Beit Haemek, Israel). This deprivation medium was supplemented with heat-inactivated dialyzed (10,000-kDa cutoff) fetal calf serum at 1% for promastigotes and 2.5% for amastigotes. Deprivation was carried out at either 26°C or 37°C for 2 h and terminated by washing twice in ice-cold Earle’s salt solution at the respective pHs. Final suspension was done according to the analyses required.

### Western blot analyses.

Western blot analysis was done as described previously (10), using a 1:1,000 dilution of rabbit anti-AAP3 N terminus antisera (11).

### Transport assays.

The uptake of 25 μM l-[^3^H]arginine (600 mCi/mmol) into axenic L. donovani promastigotes and amastigotes was determined using the rapid filtration technique as described previously (11). Briefly, the transport reaction mixture contained 1 × 10^8^ cells/ml in ice-cold Earle’s balanced salt solution at pH 7 (promastigotes) or pH 5.5 (amastigotes) supplemented with 5 mM glucose. Cells were prewarmed at either 30°C for 10 min (promastigotes) or 37°C for 5 min (amastigotes) prior to the addition of radiolabeled arginine. At 0, 0.5, 1, 1.5, 2, and 3 min, the cell suspensions were filtered through glass fiber GF/C filters that were then washed twice with ice-cold Earle’s solution.

The amount of radiolabel associated with the cells was linear with time over the 2-min time course of the assay, so the initial rate of transport was calculated from the slope of the line fitted by linear regression (see Fig. S1 in the supplemental material for promastigotes and Fig. 3B for amastigotes).

### RNA isolation and real-time quantitative reverse transcription-PCR.

RNA was isolated using Tri reagent (Sigma-Aldrich Ltd.) and a Direct-zol RNA MiniPrep kit (Zymo Research), according to the manufacturers’ instructions. Eluted RNA samples were quantified using a NanoDrop One spectrophotometer (Thermo Scientific). Two micrograms of the extracted RNA was subjected to DNase treatment using RQ1 (Promega). Successful DNase treatment was verified by PCR to make sure that no residual DNA could be responsible for amplification. cDNA was synthesized from 2 μg of DNase-treated RNA using a qScript cDNA synthesis kit (Quanta Biosciences) in a 40-μl total volume. Real-time quantitative reverse transcription-PCR (qRT-PCR) was carried out with the reagents of SsoAdvanced universal SYBR green supermix (Bio-Rad Ltd.) in a 10-μl reaction volume (5 μl SYBR green, 0.5 nM forward primers, 0.5 nM reverse primers, and 2.5 μl cDNA template) on a CFX96 Touch real-time PCR system (Bio-Rad). The AAP3 primers matched both *AAP3.1* and *AAP3.2*. Primers specific for a regulatory subunit of protein kinase A (PKAR′) were used as a control. All the samples were run in triplicates, including a no-template (negative) control for all primers used. Also, RNAseq data of the arginine deprivation response indicated that PKAR′ is not affected by the ADR, thus serving as a good control for testing LdAAP3 behavior under ADR and related conditions (Table 1).

**TABLE 1:** Primers used

Primer	Target	Amplicon size (bp)	Direction	Sequence
LinJ.31.0910	AAP3	137	Forward	5′-GGCTTCATCTTCCCTGCGTA-3′
LdBPK_3109010.1			Reverse	5′-CGGTCGAAATGGTGCCAAAC-3′

LinJ.34.2680	PKAR′	239	Forward	5′-AGACCGCGAAGCTCAGTC-3′
LDBPK_342680.1			Reverse	5′-TTGTCGCAGAGAGTACCG-3′

LinJ.10.1450	Pteridine transporter	171	Forward	5′-TGCATGTGCGGCATCTTTGGA-3′
LdBPK_101450.1			Reverse	5′-GCACAGGATAACCGAGATGC-3′

LinJ.36.2900	Nodulin-like/MFS transporter	160	Forward	5′-GTCATCGGGCTGGCCAAG-3′
LdBPK_362900.1			Reverse	5′-GAGGCAGTGCAATGAGAAGC-3′

PCR was performed at 95°C for 30 s, followed by 39 cycles of 95°C for 10 s and 60°C for 30 s; melt curve analysis was carried out at 65°C to 95°C with 0.5°C increments for 5 s/step; and data analysis was carried out as described previously (25). Briefly, *C_P_* values were obtained from all samples in triplicate (except negative controls). Primers were calibrated on pooled samples, and primer efficiencies (*E*_target_ and *E*_ref_) were calculated and incorporated into the equation below, where LdAAP3, pteridine, or MFS transporters were the target genes and PKAR′ served as the reference gene. Ratios were calculated with the *C_P_* (crosspoint) of 0-h or 48-h infected THP-1 macrophages. WT L. donovani 48-h infected macrophages served as the control.

$$\text{Ratio =}\frac{{\left(E_{\text{target}}\right)}^{\Delta C_{P}{}_{{}_{\text{target}}}\left(\text{control}-\text{sample}\right)}}{{\left(E_{\text{ref}}\right)}^{\Delta C_{P}{}_{{}_{\text{ref}}}\left(\text{control}-\text{sample}\right)}}$$

### Northern blot analyses.

Total RNAs from 5 × 10^8^ L. donovani promastigotes (before or after 2 h of arginine deprivation) or infected THP-1 cell cultures were extracted using Tri reagent (catalog no. T9424; Sigma Ltd.) and a previously described extraction protocol (9). Blots were hybridized to the CDS of LdAAP3 as described previously (9), using PCR-amplified L. donovani genomic DNA. The probe was amplified with the following primers to yield a 538-bp amplicon (LinJ.31.0900/0910 and LdBPK_310900.1/09010.1): forward primer 5′-GGCTTCATCTTCCCTGCGTA-3′ and reverse primer 5′-GTACGTCGCCAGCCAGTG-3′.

### Whole-genome sequencing.

Genomic DNA was prepared from *Leishmania* promastigotes using proteinase K digestion followed by phenol-chloroform extraction, ethanol precipitation, and fragmentation to 200 bp using a Covaris S2 sonicator according to the manufacturer’s protocol. Next-generation sequencing (NGS) libraries were prepared using a New England BioLabs (NEB) NextUltra II kit, quantified on Agilent bioanalyzer and Qubit instruments, and sequenced on an Illumina HiSeq 4000 instrument to generate 46 million to 96 million paired-end 75-bp reads, depending on the sample. Reads were aligned to the L. donovani 1S genome sequence assembled from a combination of PacBio and Illumina reads (Sur et al., unpublished) using Bowtie2 within Geneious, and the gene copy number was estimated by normalizing the read counts per gene to account for library size and assuming 4 copies of chr31.

### Infectivity assays.

THP-1 cells grown in RPMI 1640 medium with 10% FBS (catalog no. R8758) and a 1% penicillin-streptomycin solution (catalog no. 03-031-1B; Biological Industries ) in a humidified 37°C, 5% CO_2_ air atmosphere were seeded onto glass coverslips (1 × 10^6^ cells/well) in a 6-well plate and treated with 50 ng/ml of phorbol myristate acetate (PMA) (catalog no. P8139; Sigma-Aldrich, USA) for 48 h. The cells were infected as described previously (12), and the intracellular parasite load was visualized by Giemsa staining. Briefly, differentiated THP-1 macrophages were infected with log-phase L. donovani promastigotes at a multiplicity of infection (MOI) of 10 for 4 h. Parasites were washed three times in ice-cold Earle’s solution (X1) prior to infection to wash out arginine in the growth medium. Infection and subsequent incubation were performed with either 0.1 or 1.5 mM arginine in RPMI 1640 medium. Totals of 0.1 and 1.5 mM arginine in RPMI 1640 medium were prepared from no-arginine, no-leucine, and no-lysine RPMI 1640 medium (catalog no. R1780; Sigma) supplemented with 0.1 or 1.5 mM arginine, leucine (120 g/liter), lysine (70 g/liter), 10% FBS, and a 1% penicillin-streptomycin solution (catalog no. 03-031-1B; Biological Industries). Following 4 h of coincubation, the medium was aspirated from the wells. Cells were washed a total of five times with warm phosphate-buffered saline (PBS) and either collected (0-h time point) or incubated for 48 h (48-h time point) in RPMI 1640 medium containing either 0.1 mM or 1.5 mM arginine until coverslips were collected for Giemsa staining (catalog no. 32884; Sigma), and RNA was collected by direct resuspension of well contents in 600 μl Tri reagent (catalog no. T9424; Sigma).

### *In vivo* BALB/c mouse infections and analysis.

L. donovani infections were done by tail intravenous (i.v.) injection of 10^8^ stationary-phase promastigotes per mouse. On day 21 of infection, the mice were sacrificed, and mouse liver DNA was isolated using the proteinase K method (26). Quantitative PCR was performed on mouse liver DNA using mouse BDNF as the reference gene and parasite gp63 as the target gene with previously reported primer sequences (27, 28).

qPCR was carried out with the reagents of SYBR green (catalog no. A25776; Thermo Fisher Scientific) in a 10-μl reaction volume (5 μl SYBR green, final primer concentrations of 300 nM forward primer and 300 nM reverse primer, and 125 ng DNA template) on a CFX96 real-time PCR system (Bio-Rad). All samples were run in triplicate separately for the 2 primer sets, and the PCR cycle included a 30-s incubation step at 95°C and then 40 cycles of 5 s at 95°C and 30 s at 60°C. The output of normalized expression was determined using the Bio-Rad software of the instrument.

### Scanning electron microscopy images from crude liver homogenates.

Crude liver homogenates were prepared from dissected mouse livers (*n* = 6) of different cohorts of infection. The dissected mouse liver samples were crushed in the presence of Karnowsky fixative to prepare a crude liver homogenate. The homogenate was processed further with tannic acid fixation followed by 4% osmium tetroxide. The samples were serially dehydrated in ethanol and freon dried. The grids were coated with gold and visualized using an FEI Quanta 200 field emission gun (FEG) electron microscope. The samples were screened for the presence of amastigotes in 200 fields per grid.
